# Drug pair-derived synergistic therapy of flavonoids luteolin and astragaloside IV promotes neural repair following spinal cord injury via antioxidant and neuroprotective effects

**DOI:** 10.1093/pcmedi/pbaf037

**Published:** 2025-12-18

**Authors:** Wei Lin, Peng Zhang, Defeng Liu, Yihui Feng, Dongdong Su, Xin Sun, Na Yuan, Xin Zhou, Zhen Liu, Shen Liu, Huiqian Gao, Liming Li, Wenzhao Wang, Ting Tian, Jihui Zheng

**Affiliations:** Department of Orthopedics, Traditional Chinese Medicine-Western Medicine Hospital of Cangzhou, Cangzhou 061001, China; Hebei Key Laboratory of Integrated Traditional and Western Medicine in Osteoarthrosis Research, Cangzhou 061001, China; Department of Orthopedics, Traditional Chinese Medicine-Western Medicine Hospital of Cangzhou, Cangzhou 061001, China; Hebei Key Laboratory of Integrated Traditional and Western Medicine in Osteoarthrosis Research, Cangzhou 061001, China; Department of Orthopedics, Traditional Chinese Medicine-Western Medicine Hospital of Cangzhou, Cangzhou 061001, China; Hebei Key Laboratory of Integrated Traditional and Western Medicine in Osteoarthrosis Research, Cangzhou 061001, China; School of Rehabilitation Sciences and Engineering, University of Health and Rehabilitation Sciences, Qingdao 266113, China; Shandong Key Laboratory of Neurorehabilitation, University of Health and Rehabilitation Sciences, Qingdao 266113, China; Department of Orthopedics, Traditional Chinese Medicine-Western Medicine Hospital of Cangzhou, Cangzhou 061001, China; Hebei Key Laboratory of Integrated Traditional and Western Medicine in Osteoarthrosis Research, Cangzhou 061001, China; School of Rehabilitation Sciences and Engineering, University of Health and Rehabilitation Sciences, Qingdao 266113, China; Department of Orthopedics, Traditional Chinese Medicine-Western Medicine Hospital of Cangzhou, Cangzhou 061001, China; Hebei Key Laboratory of Integrated Traditional and Western Medicine in Osteoarthrosis Research, Cangzhou 061001, China; Department of Orthopedics, Traditional Chinese Medicine-Western Medicine Hospital of Cangzhou, Cangzhou 061001, China; Hebei Key Laboratory of Integrated Traditional and Western Medicine in Osteoarthrosis Research, Cangzhou 061001, China; Department of Orthopedics, Traditional Chinese Medicine-Western Medicine Hospital of Cangzhou, Cangzhou 061001, China; Hebei Key Laboratory of Integrated Traditional and Western Medicine in Osteoarthrosis Research, Cangzhou 061001, China; Department of Orthopedics, International Science and Technology Cooperation Base of Spinal Cord Injury, Tianjin Key Laboratory of Spine and Spinal Cord Injury, Tianjin Medical University General Hospital, Tianjin 300052, China; School of Rehabilitation Sciences and Engineering, University of Health and Rehabilitation Sciences, Qingdao 266113, China; School of Rehabilitation Sciences and Engineering, University of Health and Rehabilitation Sciences, Qingdao 266113, China; Department of Orthopaedics, Qilu Hospital of Shandong University, Shandong University Centre for Orthopaedics, Advanced Medical Research Institute, Cheeloo College of Medicine, Shandong University, Jinan 250012, China; School of Rehabilitation Sciences and Engineering, University of Health and Rehabilitation Sciences, Qingdao 266113, China; Department of Orthopedics, Traditional Chinese Medicine-Western Medicine Hospital of Cangzhou, Cangzhou 061001, China; Hebei Key Laboratory of Integrated Traditional and Western Medicine in Osteoarthrosis Research, Cangzhou 061001, China

**Keywords:** luteolin, astragaloside IV, traditional Chinese medicine, spinal cord injury, oxidative stress, network pharmacology

## Abstract

**Background:**

Spinal cord injury (SCI) induces a damaging oxidative microenvironment exacerbating secondary injury. The traditional Chinese medicine (TCM) drug-pair Dangshen and Huangqi, known for antioxidant and neuroprotective effects, yields key components luteolin (Lut) and astragaloside IV (AST), both promising in oxidative stress-related neurological disorders but unexplored in combination for SCI.

**Methods:**

We investigated the synergistic antioxidant effects of Lut-AST combination therapy using an *in vitro* oxidative stress model in PC12 cells, and subsequently assessed its neuroprotective efficacy through behavioral assessments and histopathological analyses in a rat model of severe SCI. Finally, we utilized network pharmacology and molecular docking to predict and explore the potential of the Lut-AST drug pair for treating SCI through multi-target therapy.

**Results:**

Our study demonstrated that the Lut-AST drug pair synergistically attenuated oxidative stress-induced cytotoxicity. Lut-AST treatment effectively promoted nerve repair and functional recovery in SCI rats. A significant recovery of motor functions was observed accompanied byh reduced accumulation of reactive oxygen species. Neuroinflammation and glial scars were largely alleviated, while the distribution of 5-hydroxytryptamine and neurofilament-positive nerve fibers was evidently increased.

**Conclusion:**

These findings confirm Lut-AST’s therapeutic efficacy in mitigating post-SCI oxidative stress and unveil novel insights into traditional Chinese medicine’s inherent multi-component synergistic interactions, suggesting potentiated outcomes through integrated antioxidant mechanisms and multi-target regulation. This study provides a paradigm for optimizing TCM-derived neuroprotective strategies by leveraging component synergy, informing novel combinatorial therapies for SCI management.

## Graphical Abstract

**Graphical Abstract:**
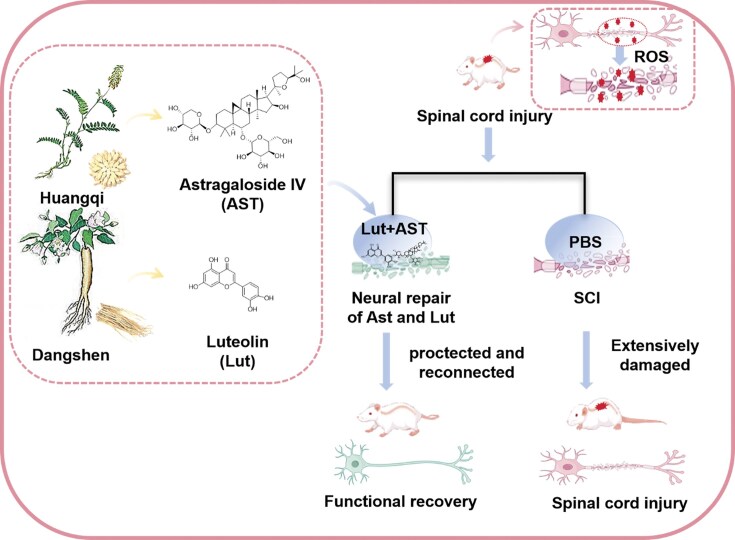
Graphical abstract illustrating that the Lut-AST combination, derived from the classic TCM herb-pair Huangqi and Dangshen, promotes functional recovery after SCI by synergistically mitigating oxidative stress and neuroinflammation.

## Introduction

Spinal cord injury (SCI) represents a devastating neurological disorder characterized by impaired neural signal transmission due to mechanical trauma or pathological damage to spinal tissues [1–3]. This condition induces severe neural tissue destruction, and affects diverse spinal segments, leading to severe motor, sensory, and autonomic dysfunction [4, 5]. The pathophysiological process involves primary mechanical injury followed by secondary degeneration cascades marked by severe oxidative stress, neuroinflammation, and excitotoxicity at the lesion site [6, 7]. These processes drive persistent neural toxicity, demyelination, glial scar formation, and ultimately cystic cavity development—pathological hallmarks that severely limit endogenous repair mechanisms [8]. Current clinical treatment following SCI mainly includes surgical decompression, methylprednisolone pulse therapy, neuroprotection, and neurorehabilitation approaches [9]. These therapeutic strategies have failed to address the multifactorial nature of SCI pathophysiology and achieved minimal functional recovery in longitudinal studies. Emerging research priorities focus on interrupting the oxidative–inflammation–apoptosis triad through neuroprotective agents [10]. However, the clinical efficacy of individual therapeutic strategies is limited by the redundancy of pathways involved in the secondary injury cascade of SCI. [11]. This therapeutic gap underscores the urgent need for multi-target interventions that can simultaneously modulate oxidative stress resolution, suppress neuroinflammation, and enhance axonal regeneration, a pharmacological paradigm in which the polypharmacology of traditional Chinese medicine (TCM) shows significant promise.

TCM compatibilities often exhibit significant synergistic effects in therapeutic applications. A two-drug compatibility is usually called a drug-pair. The compatibility of Dangshen and Huangqi is a drug-pair that is well-known for its strong antioxidant properties and neuroprotective effects. The herb Huangqi exhibits diuretic and invigorating properties, achieving dual effects of tonification and purification [12, 13]. Dangshen demonstrates dual efficacy in nourishing blood and promoting blood circulation [14]. The application of the Dangshen–Huangqi drug-pair synergistically enhances spleen-strengthening and yang qi-invigorating functions while simultaneously benefiting pulmonary nourishment and cardiac tonification [15–17]. These multi-component herbal formulations exert synergistic therapeutic effects through multiple bioactive constituents, targets, and pathways. Previous studies have demonstrated that the combination exhibits synergistic anti-aging, antioxidant, and immunomodulatory effects [18], and their co-administration enhances therapeutic potential compared to individual applications. The aqueous extracts of Dangshen and Huangqi elevate superoxide dismutase and glutathione peroxidase activities while reducing malondialdehyde levels, thereby demonstrating robust antioxidant capacity [18]. This combinatorial intervention strengthens the body's oxidative defense mechanisms [19]. The Dangshen–Huangqi formula holds significant therapeutic value in neural diseases, with evidence supporting its synergistic enhancement of cognitive function. Preclinical studies have confirmed that the Dangshen–Huangqi herb pair exerts synergistic effects in enhancing antioxidant capacity, immune regulation, cognitive function, and anti-aging properties. However, the specific contributions of its key components luteolin (Lut) and astragaloside IV (AST) to SCI repair remained unaddressed. This gap motivated our focus on deconstructing the herb pair to validate the efficacy of these two components.

Lut is a flavonoid with well-documented antioxidant and anti-inflammatory effects, primarily via activating the Nrf2/HO-1 (Nuclear factor erythroid 2–related factor 2 - Heme Oxygenase-1) pathway and inhibiting NF-κB signaling [20–24]. AST, a triterpenoid saponin, exhibits robust neuroprotective properties, including promoting axonal regeneration, enhancing neurotrophic factor secretion, and scavenging reactive oxygen species (ROS) [25–28]. Their complementary mechanisms including antioxidant and neuroregenerative effects, which align with the multifactorial pathophysiology of SCI, make them a logical combinatorial candidate. Single-target synthetic drugs (e.g. methylprednisolone) only target inflammation but have systemic toxicity [29, 30]. The therapeutic efficacy of individual natural compounds is often limited. For instance, the natural compound Lut alone lacks direct neuroregenerative effects [23], while AST has weaker ROS-scavenging capacity [31]. In contrast, the Lut-AST combination offers a distinct advantage through its multi-target, complementary mechanism of action. Lut-AST could simultaneously modulate three core pathological processes of SCI by inhibiting neuroinflammation, eliminating oxidative stress, and suppressing scar formation, thus promoting the repair of SCI.

We hypothesize that the combination of Lut and AST exerts synergistic therapeutic effects in SCI by: (i) mitigating oxidative stress via complementary antioxidant mechanisms (Lut: Nrf2 activation; AST: direct ROS scavenging); (ii) suppressing neuroinflammation by inhibiting microglial activation and NF-κB/TNF-α signaling; and (iii) promoting neural repair by reducing glial scar formation and enhancing axonal regeneration [via 5-hydroxytryptamine (5-HT) and neurofilament (NF) upregulation]. This synergy would ultimately improve motor function recovery more effectively than either component alone. To validate this potential, we made evaluations in an *in vitro* oxidative stress model and an *in vivo* severe SCI rat model. The results elucidated the mechanistic roles of Lut-AST in oxidative stress regulation following SCI, potentially advancing novel therapeutic strategies for injury management and treatment optimization.

## Materials and methods

### Cell culture and drug application

PC12 cells were cultured in 1640 basal medium (Procell) containing 10% fetal bovine serum (PAN) and 1% penicillin/streptomycin (Procell). Hydrogen peroxide solution was applied at an appropriate concentration for 24 h to simulate the H_2_O_2_ species damage to neurons. Lut-AST was dissolved in dimethyl sulfoxide (DMSO, Solarbio), with the final concentration of DMSO being < 0.1% (volume/volume).

### Cell viability assay

PC12 cells in the logarithmic growth phase were seeded into 96-well plates at a density of 8000 cells/well, with 100 μl of cell suspension in each well. After the cells adhered, different concentrations of H_2_O_2_ and Lut-AST were added to stimulate the cells for 24 h. The original medium was aspirated, and CCK-8 (Cell Counting Kit-8) (Biosharp) working solution (medium:CCK-8 solution = 9:1) was added. The cells were incubated in a 37°C incubator in the dark for 1 h, and the absorbance value of each well at 450 nm was detected using a microplate reader. Cell survival rate = [(*A*s−*A*b)/(*A*c−*A*b)] × 100%, where As is the absorbance of the sample well, Ac is the absorbance of the control well (cells without treatment), and Ab is the absorbance of the blank well (medium without cells)..

### ROS assay

PC12 cells were cultured in 96-well plates overnight, then treated with H_2_O_2_ (40 μM) alone or with Lut (5 μM), AST (20 μM), and Lut-AST (5/20 μM) for 24 h. Subsequently, the cells were treated with 2’,7’-dichlorodihydrofluorescein diacetate (DCFH-DA) and Hoechst 33 342 (1 μg/ml) and incubated for 20 min. After washing with phosphate-buffered saline (PBS), images were acquired using a fluorescence microscope. Intracellular ROS levels were assessed by measuring the relative fluorescence intensity of DCFH-DA.

### Animal model

Female SD rats (weighing 220 ± 10 g) were procured from Beijing HFK Bioscience Co. Ltd (Beijing, China). Animals were housed under controlled conditions (24 ± 1°C, 55% ± 5% humidity, 12-h light/dark cycle) with *ad libitum* access to standard feed and autoclaved water. All surviving animals completed the entire experimental protocol. The animal procedures were approved by the Science and Technology Ethics Committee of the University of Health and Rehabilitation Sciences (approval No. KFDX-2025–1049) on 28 February 2025. Female rats were selected based on the following reasons. After SCI, rats lose autonomous urinary function and rely on manual-assisted urination for urine excretion. Female rats have a short, wide, and straight urethra that directly connects the urinary bladder to the external urethral orifice. This anatomical feature allows urine to be smoothly discharged by bladder compression, resulting in low nursing difficulty and a relatively low risk of urinary tract infection. In contrast, male rats possess a long, narrow, and highly curved urethra. Even with bladder compression, complete emptying of urine remains challenging, which easily leads to urinary retention and recurrent urinary tract infections. These complications significantly compromise the stability of the SCI model and reduce the survival rate of male rats. Based on anatomical characteristics and practical nursing feasibility, female rats are more suitable as experimental subjects for the SCI model.

To ensure a sufficient number of animals for final analysis, a total of 21 rats were initially allocated to the three experimental groups: sham (*n* = 6), SCI (*n* = 8), and SCI + Lut-AST (*n* = 7). The SCI model was established as previously described [32]. Briefly, following anesthesia and dorsal hair shaving, the rats underwent aseptic preparation at the surgical site. A laminectomy was performed at the T7-T8 vertebral level to fully expose the spinal cord. The spinal cord was then completely transected using micro-scissors to create a 5 mm lesion gap. The SCI group received a single *in situ* injection of 10 μl of DMSO vehicle at the injury epicenter, while the Lut-AST group received a single *in situ* injection of the Lut-AST co-formulation (Lut: 2.5 μg/kg, AST: 5 μg/kg) dissolved in DMSO. The sham group received anesthesia and underwent laminectomy without spinal cord transection or injection. Subsequently, the paravertebral musculature and overlying skin were sutured in layers to close the incision. Following surgery, rats received intraperitoneal injection of penicillin (50 000 U/100 g/day) for 1 week to prevent infection. Bladder expression was performed twice daily until spontaneous voiding function was restored. During the post-operative period, two rats in the SCI group and one rat in the Lut-AST group died, resulting in an overall survival rate of 85.7%. Consequently, the final analysis included 6 rats in each group (total *n* = 18), as indicated in the figure legends. Basso Beattie Bresnahan (BBB) scoring was conducted at 1 day post-surgery and weekly thereafter. At 5 weeks post-surgery, animals underwent horizontal-ladder testing followed by BBB scoring. Subsequently, transcardial perfusion and tissue collection were performed for histological staining.

### Hematoxylin-eosin staining

The heart, liver, spleen, lung, and kidney tissues of experimental rats were processed in a series of steps: first, they were treated with xylene twice, then with absolute ethanol twice, followed by dehydration through 95%, 80%, and 70% ethanol gradients, and finally rehydrated with deionized water to complete the dewaxing and rehydration process. The tissue sections were first stained with hematoxylin solution for 5 min for nuclear staining, then differentiated by brief immersion in acid alcohol (70% ethanol containing 1% hydrochloric acid) five times, and finally thoroughly rinsed with distilled water; then stained with eosin solution for 3 min to complete cytoplasmic counterstaining, followed by dehydration through a series of ethanol gradients, cleared with xylene, and finally sealed with neutral gum.

### Immunofluorescence staining

The spinal cord was subjected to immunofluorescent staining, followed by embedding, and cryo-sectioned into 16 μm sections. The sections were then stained with primary antibodies including: NF (Cell Signaling Technology, USA), glial fibrillary acidic protein (GFAP; Boster, China), ionized calcium-binding adaptor molecule 1 antibody (Iba1; WAKO, Japan), and 5-HT (WAKO, Japan), and incubated overnight at 4°C. After washing, the tissue sections were incubated for 1 h at 37°C with Alexa Fluor 488 and 594 conjugated secondary antibodies (Proteintech, China). Subsequently, the sections were mounted using an anti-fade mounting medium (Solarbio). The samples were imaged using a 3DHISTECH sweeper (Hungary). Quantitative analysis was performed using ImageJ software.

### DHE staining

Sections were rinsed in PBS for 10 min to remove residual optimal cutting temperature (OCT). Target tissue areas were demarcated using an immunohistochemical barrier pen. A 5 μm dihydroethidium (DHE) working solution (Solarbio) in PBS was applied to completely cover the sections, followed by dark incubation at 37 °C for 60 min. Finally, sections were PBS-washed to remove unreacted dye. The samples were imaged using a 3DHISTECH sweeper (Hungary). Quantitative analysis was performed using ImageJ software.

### BBB scores

BBB scores were used to evaluate the hindlimb motor function of the rats. The rats were allowed to crawl freely. The range of motion of the three joints in the hindlimbs, the movement posture, and the coordination between the hindlimbs and the forelimbs of the rats were observed. Each blinded observer independently observed for 4 min, and the hindlimb motor function of the rats was scored according to the BBB rating scale (supplementary Table 1, see online supplementary material). Relevant images depicting the SCI modeling procedure, tissue sampling, and BBB scoring process have been provided in the online supplementary  materials.

### Ladder climbing experiment

The horizontal ladder test is a widely adopted assessment method in this field, designed to analyze motor function and skilled walking ability in laboratory rodents. In this experiment, animals walked on a horizontal ladder with adjustable rung intervals, which challenges their adaptability while simultaneously measuring the functional status of both forelimbs and hindlimbs. Particular attention is given to the functional status of forelimbs and hindlimbs when assessing skilled walking ability in rats. The evaluation covers multiple parameters including limb placement, stepping patterns, and coordination, ensuring a comprehensive qualitative and quantitative analysis.

### Reagents

All chemical reagents were of analytical grade. The specifics of all commercial reagents, including antibodies, kits, and critical chemicals, are comprehensively listed in supplementary Table 2, see online supplementary material.

### Network pharmacology analysis

The 2D and 3D molecular structures and simplified molecular input line entry system (SMILES) notations of Lut and AST were downloaded from the PubChem database (https://pubchem.ncbi.nlm.nih.gov/). Target prediction was performed using the TCMSP database (http://tcmspw.com/tcmsp.php), SwissTargetPrediction database (http://www.swisstargetprediction.ch/), and PharmMapper databases (http://www.lilab-ecust.cn/pharmmapper/). SCI-related targets were retrieved from the GeneCards database (https://www.genecards.org/), OMIM database (https://www.omim.org/), and DisGeNET database (https://www.disgenet.org/) [33]. The UniProt IDs of these targets were searched using the UniProt database (https://www.uniprot.org), with the species defined as “Homo sapiens”, and targets not meeting the screening criteria were excluded.

Using the bioinformatics platform (http://www.bioinformatics.com.cn/), the intersection targets between the compounds and the disease were obtained. These intersection targets were then imported into the STRING database (https://string-db.org/cgi/input.pl), the species was set to “Homo sapiens” with a minimum required interaction confidence score ≥ 0.400, and disconnected nodes were hidden to generate protein–protein interaction (PPI) relationship files. These PPI files were subsequently imported into Cytoscape software (3.9.1) for topological analysis to construct the PPI network of the intersection targets. The CytoHubba plugin was employed to identify the top 10 core hub targets by calculating the weight of each gene. Furthermore, the intersection targets were subjected to in-depth analysis using the Oebiotech Platform (https://cloud.oebiotech.com/#/bio/tools) to elucidate the key signaling pathways and biological processes involved in the therapeutic effects of Lut and AST on SCI.

### Molecular docking

The 3D structures of core targets were downloaded from the PDB database (https://www.rcsb.org/), followed by format conversion of the 3D structures of components and targets using Open Babel GUI software. Subsequently, Autodock Tools (1.5.7) were employed for processing and docking, and PyMOL (2.2.0) was utilized for 3D visualization.

### Statistical analysis

Statistical analyses were performed using GraphPad Prism version 9.0. Data are presented as mean ± SEM. Comparisons between two groups were analyzed using the unpaired Student’s t-test. For comparisons among multiple groups, one-way analysis of variance (ANOVA) was employed, followed by Tukey’s *post hoc* test for multiple comparisons. The BBB scoring, which was evaluated over time across different groups, was analyzed using two-way ANOVA with *post hoc* Bonferroni test. Significance was determined as *P < 0.05, **P < 0.01, ***P < 0.001, and ****P < 0.0001.

## Results

### Lut-AST alleviated oxidative stress damage in H_2_O_2_-treated PC12 cells

**Figure 1:**
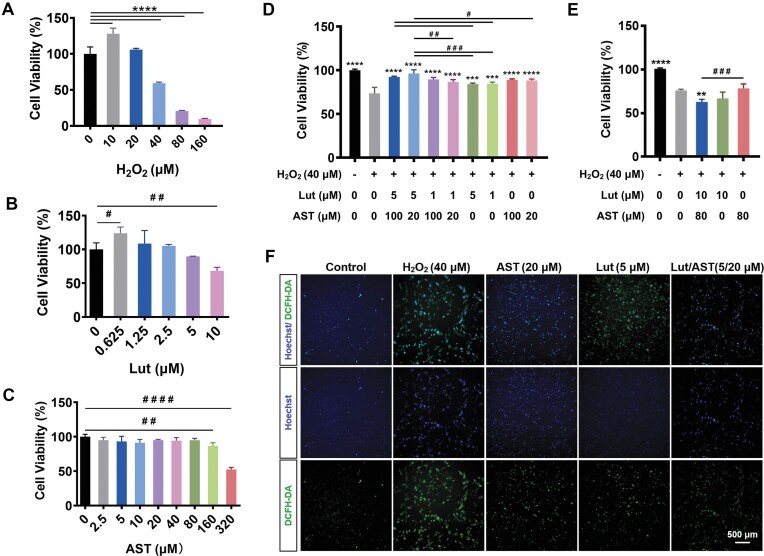
Lut-AST alleviated oxidative stress damage in H_2_O_2_-treated PC12 cells. (A) Dose-dependent effects of H_2_O_2_ (0–160 μM) on PC12 cell viability after 24-h exposure. (B) Impact of Lut (0–10 μM) on cellular viability following 24-h treatment. (C) Influence of AST (0–320 μM) on cell viability after 24-h administration. (D, E) Impact of 40 μM H_2_O_2_ and varying concentration ratios of Lut-AST on PC12 cell viability after 24-h treatment. (F) DCFH-DA fluorescent staining after 24-h H_2_O_2_ treatment with (without) Lut-AST or its individual monomers. Data are presented as mean ± SEM (*n* = 3). ****P < 0.0001 H_2_O_2_-treated group vs blank control in (A), ^##^*P* < 0.01 blank control vs AST group (160 μM), ^####^*P* < 0.0001 blank control vs AST group (320 μM) in (C) .**P < 0.01, ***P < 0.001, and ****P < 0.0001 vs H_2_O_2_-treated control group in (D, E). ^#^*P* < 0.05; ^##^*P* < 0.01; ^###^*P* < 0.001 indicate significant differences between the Lut-AST combination and its individual monotherapy groups in (D, E). Statistical analysis was performed using one-way ANOVA followed by Tukey’s *post hoc* test.

To evaluate the cytoprotective efficacy of Lut-AST co-administration against oxidative injury in PC12 neuronal models, we established a H_2_O_2_-induced oxidative stress model with exogenous Lut-AST intervention. Initial dose optimization revealed that 40 μM H_2_O_2_ exposure for 24 h induced pharmacologically relevant oxidative damage, reducing cellular viability to 50%–60% as quantified by CCK-8 assay (Fig. 1A). Therefore, this concentration was selected for subsequent CCK-8 assays (Fig. 1A). Dose–response test showed that Lut < 10 µM and AST ≤ 160 µM exhibited no cytotoxicity (Fig.1B, C). Based on these pharmacodynamic parameters, 1 and 5 µM Lut, combined with 20 and 100 µM AST, were selected for further experiments.

To investigate the synergistic protective interaction between Lut and AST against H_2_O_2_-induced oxidative stress, we assessed cytoprotective effects using a 40 µM H_2_O_2_ oxidative damage model combined with graded Lut-AST co-treatment. All combination regimens demonstrated significantly higher cellular viability compared to H_2_O_2_-exposed controls. Notably, while the combination of 1 µM Lut with either 20 or 100 µM AST failed to exhibit additive protection, the 5 µM Lut + 20 µM AST co-treatment group showed statistically superior viability compared to monotherapy groups and H_2_O_2_ controls (Fig. 1D). However, at the higher concentration of 10 µM Lut combined with 80 µM AST, a decline in cell viability was observed, potentially attributed to cytotoxic effects at these elevated doses (Fig. 1E). In addition, assessment of antioxidant activity using the DCFH-DA probe revealed that the Lut-AST combination effectively attenuated H_2_O_2_-induced ROS accumulation in PC12 cells (Fig. 1F). These findings demonstrated a concentration-dependent synergistic protective effect of Lut-AST coadministration against H_2_O_2_-mediated oxidative damage in PC12 cells, highlighting the therapeutic potential of this combined pharmacological strategy for combating ROS-induced cellular injury.

### Lut-AST promoted motor functional recovery and tissue repair after SCI

**Figure 2:**
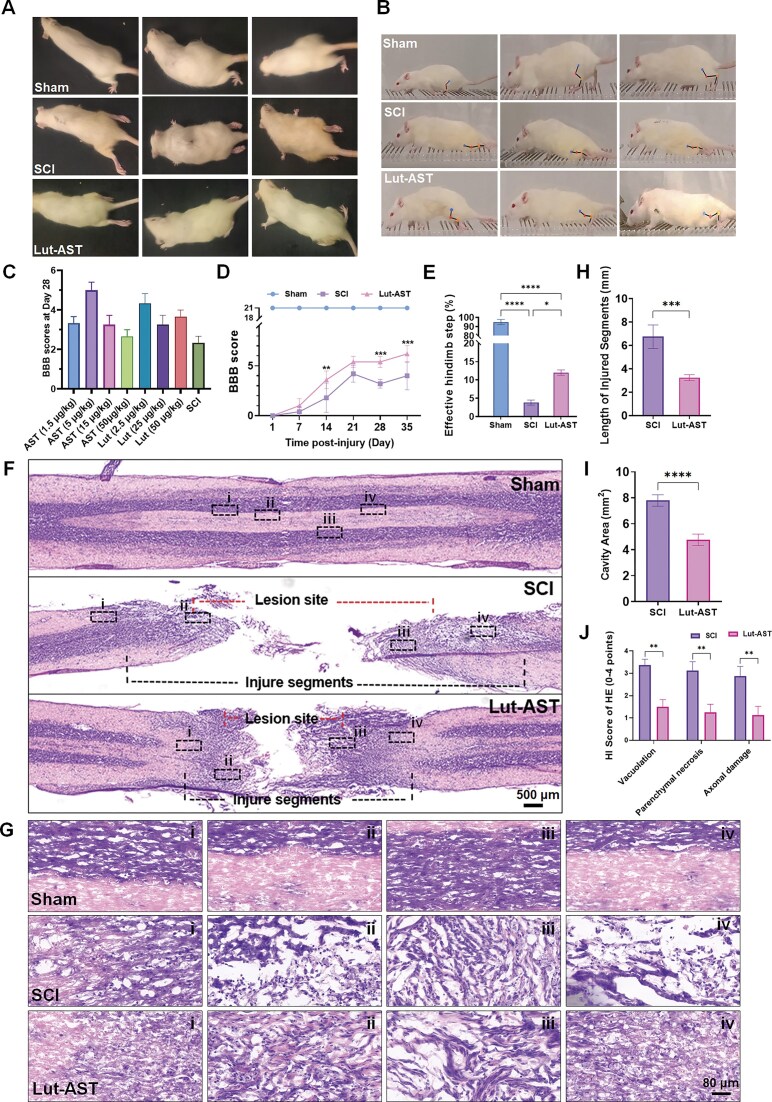
Lut-AST promoted motor functional recovery and tissue repair after SCI. (A) Representative captured images of hindlimb activity in the open field test at 35 days post-SCI. (B) Representative images of rats performing the horizontal-ladder rung walking test at 35 days post-SCI. (C) BBB scores at 28 days post-SCI for monotherapy groups treated with different concentrations of AST or Lut alone. (D) Quantitative analysis of BBB scores over 35 days post-SCI. (E) Statistical analysis of effective hindlimb steps in the horizontal-ladder test at 35 days post-SCI. (F) Longitudinal hematoxylin–eosin (HE) staining of the injury epicenter at 35 days post-SCI. (G) Higher magnification view of the HE staining in (F). (H, I) Quantitative analysis of the injured segment length (H) and lesion cavity area (I) from HE staining in (F). (J) Histopathological injury (HI) score quantification of HE staining in the injury core region. Data are shown as mean ± SEM (*n* = 6 independent samples for each group), * *P* < 0.05, ** *P* < 0.01, *** *P* < 0.001, **** *P* < 0.0001. Two-way ANOVA with *post hoc* Bonferroni test in (D), one-way ANOVA followed by Tukey’s *post hoc* test in (E), and unpaired t-test in (H–J).

To determine the optimal *in vivo* dose of Lut-AST, we first evaluated the therapeutic efficacy of individual Lut and AST monotherapies at varying concentrations following SCI. We evaluated different concentrations of Lut (2.5, 25, and 50 μg/kg) and AST (1.5, 5, 15, and 50 μg/kg) individually. BBB scoring at 28 days post-SCI indicated that Lut at 2.5 μg/kg and AST at 5 μg/kg provided the most favorable motor functional recovery. Consequently, the combination of Lut (2.5 μg/kg) and AST (5 μg/kg) was selected for all subsequent *in vivo* experiments. To quantitatively assess locomotor recovery trajectories following SCI, we performed BBB open-field testing at predefined intervals (days 1, 7, 14, 21, 28, and 35 post-SCI) using a double-blind scoring protocol. As anticipated, intact rats maintained maximal BBB scores (BBB = 21), while SCI-vehicle controls exhibited complete hindlimb paralysis (BBB = 0) within 24 h post-injury. Lut-AST combination therapy demonstrated progressive functional recovery, with statistically significant improvements compared to the SCI control group beginning at weeks 2 to 5 post-injury (Fig. 2A, D). Notably, the Lut-AST group achieved superior BBB scores relative to the SCI group at critical recovery timepoints (*P* = 0.0063 at week 2, *P* = 0.0006 at weeks 4 and 5, Lut-AST vs SCI control group), suggesting accelerated neurological rehabilitation following combinatorial treatment. In addition, during the ladder-rung walking task, the Lut-AST treatment group showed a 215.2 ± 18.6% increase in effective steps relative to the SCI group (Fig. 2B and E).

To further evaluate the therapeutic effect of Lut-AST combination post-SCI, we conducted histopathological analyses using hematoxylin and eosin (HE) staining at 5 weeks post-injury. HE staining revealed SCI pathology in the control group, including disrupted axonal architecture with disorganized neural fiber alignment and pronounced inflammatory infiltration. In contrast, the Lut-AST-treated group exhibited preserved tissue integrity, with marked reduction in both structural disorganization and leukocyte infiltration compared to the controls (Fig. 2F–J). Lut-AST treatment led to a significant reduction in the length of injured segments by 51.8 ± 8.0%, and in cavity area by 38.9 ± 4.9%, relative to the SCI group (Fig. 2H, I). Furthermore, histopathological injury (HI) scoring revealed markedly decreased scores in the Lut-AST group compared to the SCI controls, specifically in vacuolation (*P* = 0.0026), parenchymal necrosis (*P* = 0.0026), and axonal damage (*P* = 0.0052) (Fig. 2J). These histological improvements correlated with functional recovery, collectively demonstrating Lut-AST’s dual function in modulating neuroinflammation and facilitating structural reorganization within the injured spinal parenchyma.

### Lut-AST promoted nerve tissue repair after SCI

**Figure 3:**
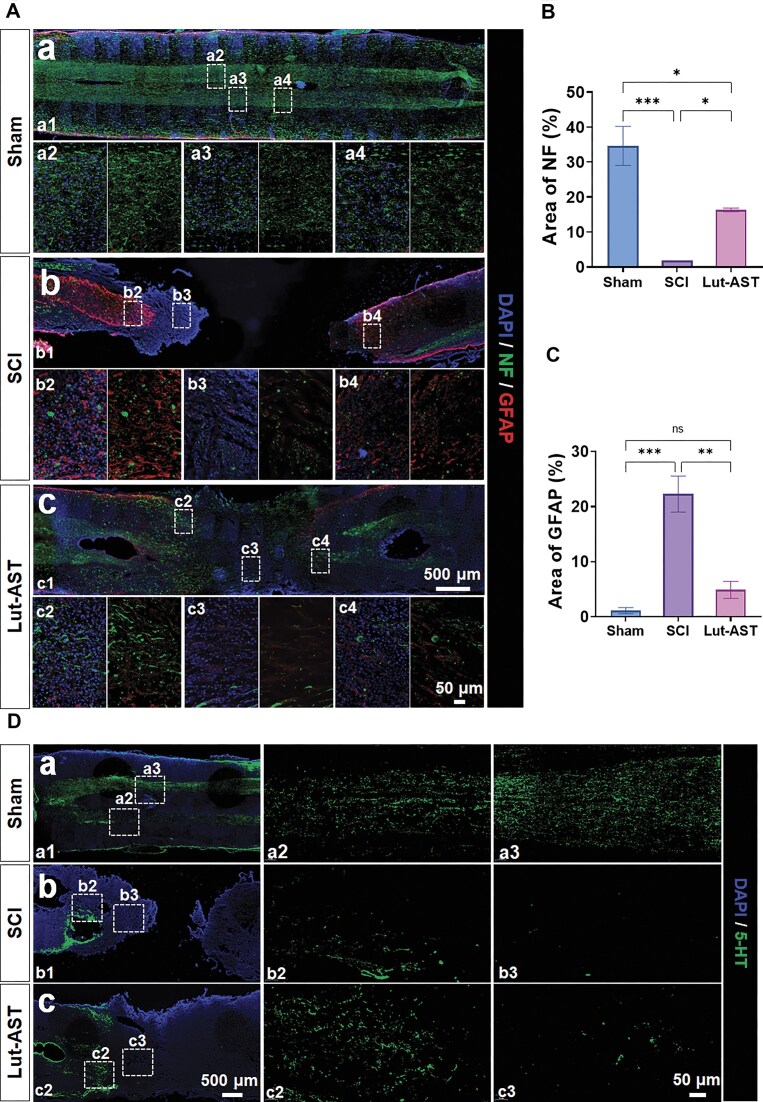
Lut-AST promoted regrowth of NF-positive neural fibers across the glial scar and 5-HT expression following SCI. (A) Double-labeled immunofluorescence images of NF (green) and GFAP (red) in longitudinal sections surrounding the injury site of rats in the Lut-AST treatment group and SCI group at 5 weeks post-injury, with corresponding quantitative analysis shown in (B) and (C). (D) Representative images showing 5-HT (green) staining in spinal cord longitudinal sections around the injury lesion of Lut-AST-treated rats and SCI rats at 5 weeks after SCI. Data are shown as mean ± SEM (*n* = 6), * *P* < 0.05, ** *P* < 0.01, *** *P* < 0.001, one-way ANOVA followed by Tukey’s *post hoc* test.

The effect of Lut-AST on nerve repair was evaluated by immunofluorescence analysis. The results showed that the staining area of NF protein at the lesion site in the injury group was significantly reduced compared to the sham group. However, Lut-AST treatment reversed this change, resulting in an 830.6 ± 30.2% increase in the NF-positive area compared to the SCI group (Fig. 3A and B). Additionally, the immunofluorescence intensity of GFAP was significantly enhanced in the SCI group, indicating the formation of glial scar. Lut-AST effectively inhibited GFAP expression, as evidenced by a 78.1 ± 8.6% reduction in the GFAP-positive area compared to the SCI group (Fig. 3A and C). These results suggest that Lut-AST treatment promoted tissue repair and inhibited the formation of glial scars.

Immunohistochemical analysis of 5-HT, a crucial neurotransmitter in spinal cord neural pathways, demonstrated significantly enhanced expression levels in the combination treatment group compared to the SCI controls. Notably, the combined Lut-AST therapy not only improved 5-HT distribution at lesion sites but also restored its expression pattern in adjacent regions of the injured spinal tissue (Fig. 3D). These findings suggest that Lut-AST combination therapy promoted functional recovery through dual mechanisms: structural restoration of serotonergic fibers and enhancement of intrinsic spinal cord repair capacity. The spatial extension of 5-HT expression beyond the lesion site underscored its potential to drive distributed neural network reorganization, a critical factor in neurological rehabilitation after SCI.

### Lut-AST reduced neuroinflammation

**Figure 4:**
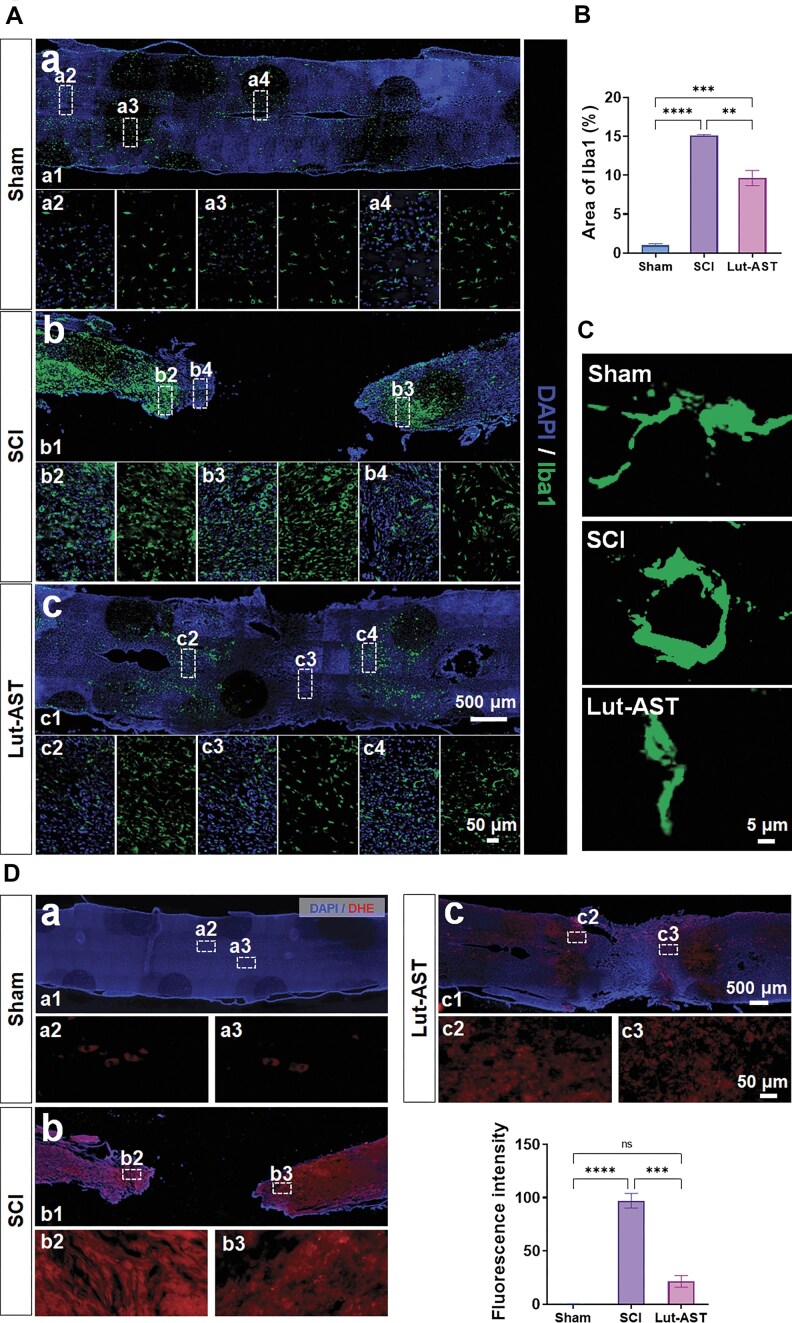
Lut-AST reduced neuroinflammation and oxidative stress after SCI. Representative Iba-1 immunofluorescence images (A) and quantitative analysis (B) in longitudinal sections around the injury core of rats from the Lut-AST group and the SCI group 5 weeks after injury. (C) Morphological changes in Iba1-positive microglia in the Lut-AST group and the SCI group 5 weeks after injury. (D) Representative DHE immunofluorescence images of longitudinal spinal cord sections. Data are shown as mean ± SEM, *n* = 6 rats in each group. ** *P* < 0.01, *** *P* < 0.001, **** *P* < 0.0001, ns, not significant, one-way ANOVA followed by Tukey’s *post hoc* test.

To evaluate the immunomodulatory capacity of Lut-AST in SCI pathophysiology, we quantified microglial activation using Iba1 immunostaining. The immunofluorescence staining revealed that the Iba1^+^ microglial area was significantly reduced in the Lut-AST group compared to SCI controls. Following SCI, microglia become overactivated, accompanied by a substantial increase in the microglial area. Conversely, Lut-AST treatment significantly suppressed this microglial activation, reducing the Iba1-positive area by 36.2 ± 6.0% compared to the SCI group (Fig. 4A and B). This finding suggested that Lut-AST possessed the ability to suppress neuroinflammation following SCI, potentially creating a permissive microenvironment for nerve regeneration. After SCI, Iba1^+^ microglia typically transitioned from a resting state to an activated state, shifting from microglia with elongated processes and smaller cell bodies to those with enlarged cell bodies and a more amoeboid-like morphology (Fig. 4C). Following Lut-AST combination therapy, Iba1^+^ microglia exhibited morphological features closer to the resting state, suggesting that the combined treatment alleviated SCI-induced inflammatory responses by modulating microglial activation.

### Lut-AST alleviated oxidative stress damage after SCI

Dihydroethidium (DHE) fluorescence intensity served as a quantitative indicator of intracellular ROS production, with signal magnitude directly proportional to oxidative stress levels. Confocal microscopy analysis revealed pronounced nuclear-localized DHE fluorescence in the SCI lesion epicenter compared to that in sham controls (Fig. 4D). Lut-AST combination attenuated the oxidative burst, and the fluorescence intensity of DHE was markedly reduced by 77.9 ± 12.6% compared to the SCI group (Fig. 4D).

### Lut-AST treatment did not induce an obvious toxicity effect in major organs

**Figure 5:**
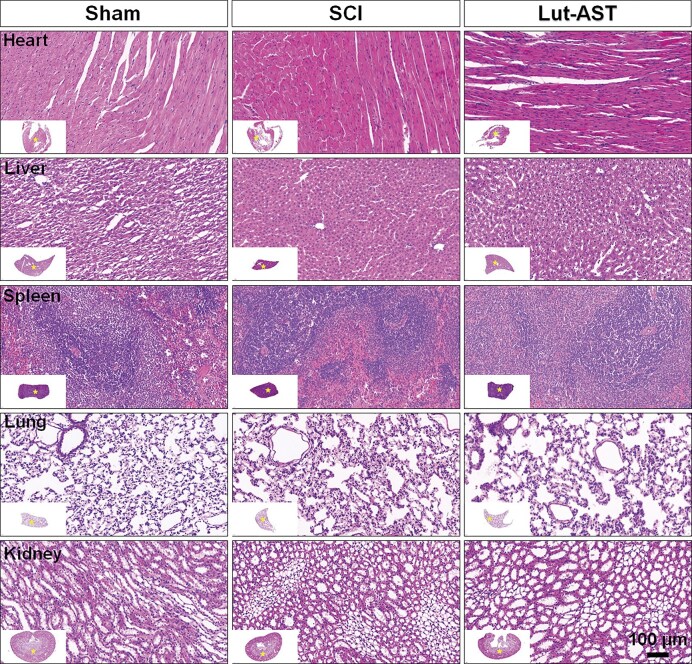
HE staining of major organ tissues across different groups.

For evaluation of the *in vivo* biocompatibility of the drug-pair, tissues of heart, lung, liver, kidney, and spleen were subjected to HE staining at 5 weeks. The results revealed no significant difference across different groups (Fig. 5). This indicated that the Lut-AST combination demonstrated excellent biological safety.

### Network pharmacology analysis

**Figure 6:**
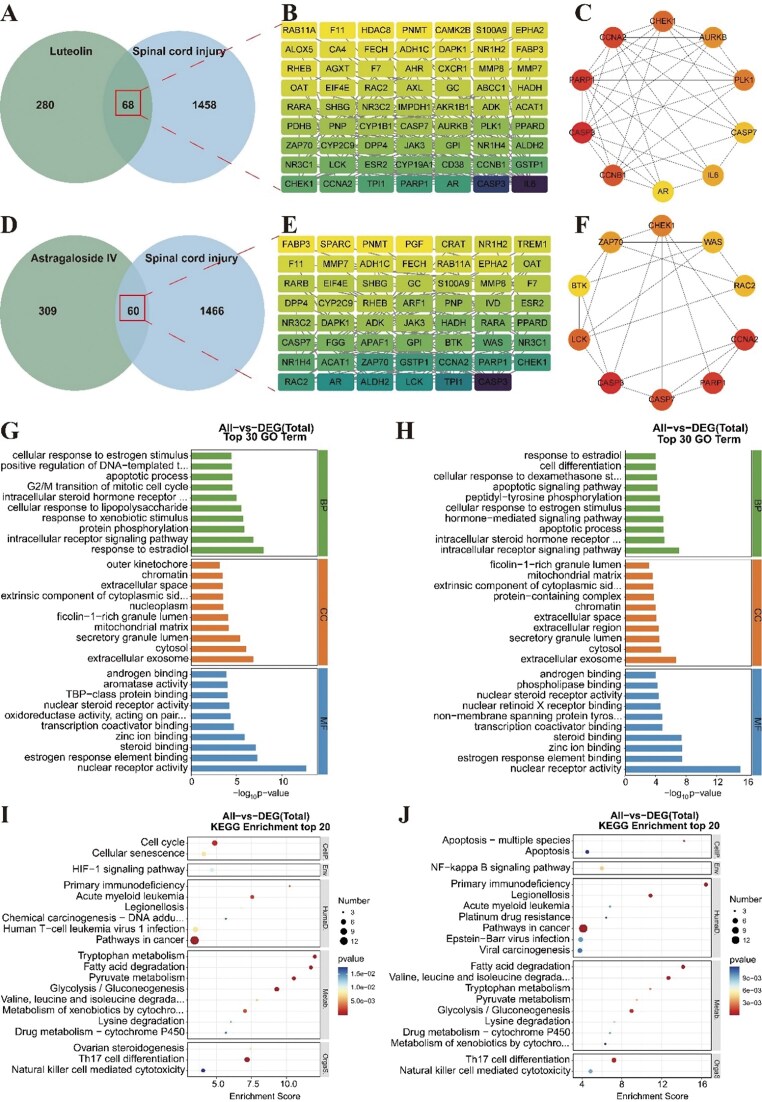
Network pharmacology analysis of Lut and AST in treating SCI. (A) Intersection targets of Lut and SCI. (B) PPI network diagram of Lut and SCI intersection targets. (C) Top 10 hub genes of Lut and SCI. (D) Intersection targets of Ast and SCI. (E) PPI network diagram of Ast and SCI intersection targets. (F) Top 10 hub genes of Ast and SCI. (G) Top 30 GO analysis results of Lut and SCI. (H) Top 30 KEGG analysis results of Lut and SCI. (I) Top 30 GO analysis results of Ast and SCI. (J) Top 30 KEGG analysis results of Ast and SCI.

A total of 1526 SCI-related targets were identified. Lut was associated with 348 targets, and Lut and SCI had 68 intersection targets (Fig. 6A). A PPI network comprising 63 nodes and 183 edges was constructed (Fig. 6B). The top 10 core targets identified by CytoHubba were AR, AURKB, CHEK1, CCNA2, PLK1, CASP3, CASP7, IL6, PARP1, and CCNB1 (Fig. 6C). AST was associated with 369 targets, AST and SCI had 60 intersection targets (Fig. 6D). As shown in Fig. 6E, the PPI network comprised 55 nodes and 121 edges. The top 10 core targets identified from this network were CASP3, CHEK1, PARP1, BTK, CASP7, LCK, CCNA2, RAC2, ZAP70, and WAS (Fig. 6F).

GO (Gene Ontology) and KEGG (Kyoto Encyclopedia of Genes and Genomes) pathway enrichment analysis further systematically elucidated the potential mechanisms and biological processes of Lut and AST in treating SCI. The top 30 enriched terms are shown in Fig. 6G–J. GO enrichment indicated that both compounds may act on common cellular components including the extracellular exosome, cytosol, secretory granule lumen, and mitochondrial matrix. They were primarily involved in biological processes related to estradiol, estrogen, and steroid hormones, and affected molecular functions such as zinc-ion binding, transcription coactivator binding, and nuclear steroid receptor activity. KEGG pathway enrichment revealed that both compounds were implicated in signaling pathways like Th17 cell differentiation and natural killer cell mediated cytotoxicity, participated in substance metabolism including fatty acid degradation, glycolysis/gluconeogenesis, and tryptophan metabolism, and were involved in environmental information processing via pathways such as the NF-κB signaling pathway. Both were also associated with processes like apoptosis—multiple species and linked to various human diseases, including pathways in cancer, primary immunodeficiency, viral carcinogenesis, and acute myeloid leukemia.

### Molecular docking

**Figure 7:**
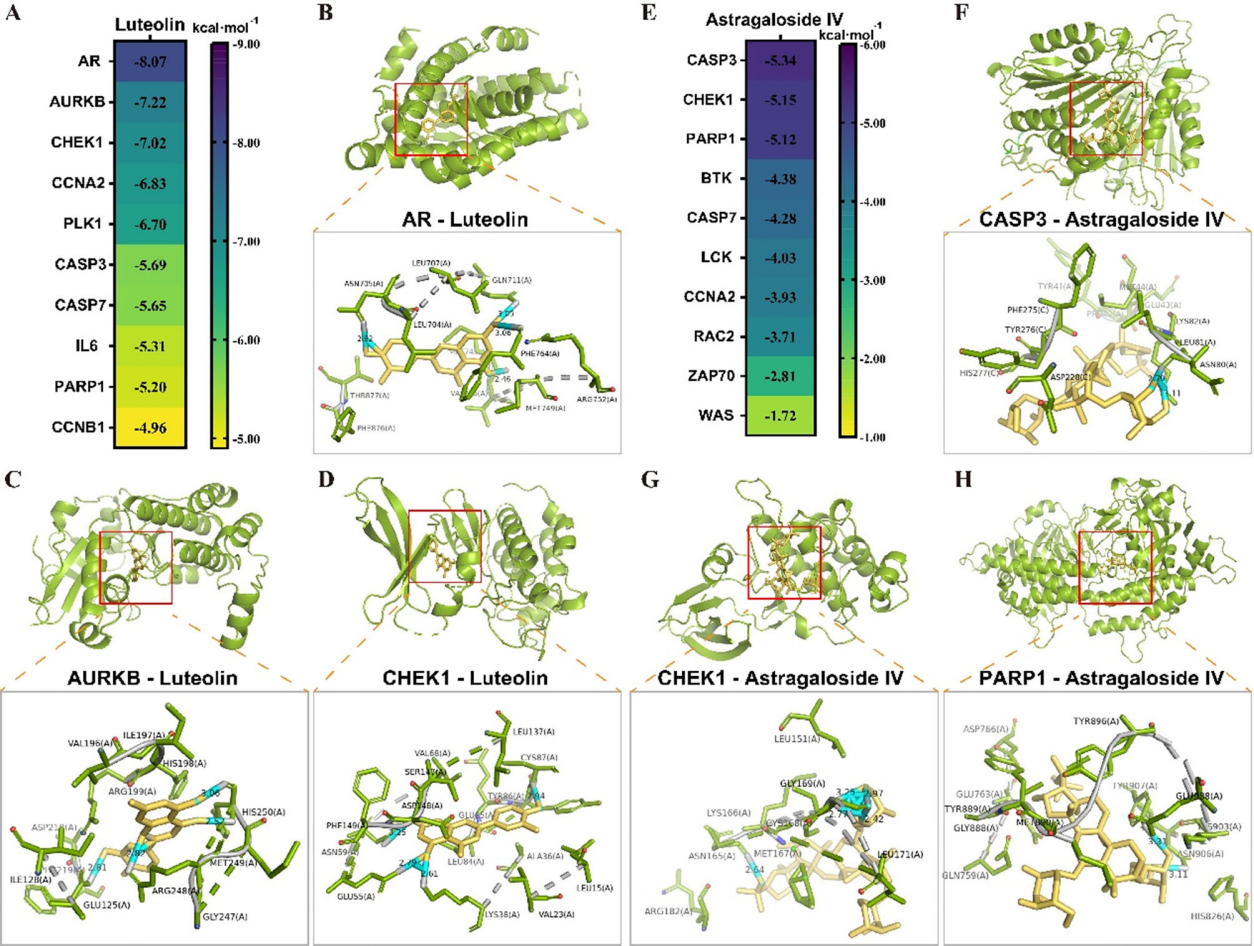
Binding status of Lut and AST with core targets. (A) Heat map of the binding energy of LUT to each protein. (B–D) Molecular docking results of LUT and AR, AURKB and CHEK1. (E) Heat map of the binding energy of AST to each protein. (F–H) Molecular docking results of AST and CASP3, CHEK1, and PARPK1.

The binding energies of Lut and AST with core target proteins are shown in Figs. 7A and 7E, respectively. Lut exhibits strong binding affinity with AR, AURKB, and CHEK1, and moderate binding affinity with CCNA2, PLK1, CASP3, CASP7, IL6, and PARP1. AST shows moderate binding affinity with CASP3, CHEK1, PARP1. Visualization of the three strongest binding results is shown in Figs. 7B–D and F–H.

## Discussion

SCI triggers excessive ROS accumulation, driving oxidative stress that exacerbates secondary neuronal apoptosis, ferroptosis, and mitochondrial dysfunction [34, 35]. Current clinical strategies, such as surgical decompression combined with high-dose methylprednisolone, remain limited by systemic toxicity and incomplete functional recovery [36–39]. In contrast, bioactive components derived from TCM, including AST and Lut, exhibit multi-target therapeutic potential by modulating oxidative stress and inflammatory cascades [40–42]. In this study, we investigated the synergistic therapeutic effects of Lut-AST combination on SCI recovery, focusing on their antioxidant and neuroprotective mechanisms. Our findings demonstrate that the Lut-AST combination significantly attenuates oxidative stress, reduces neuroinflammation, and promotes functional recovery in a rat model of severe SCI.

### Therapeutic efficacy and synergistic mechanisms of Lut-AST combination in SCI

Our *in vivo* experiments revealed that Lut-AST treatment confers significant neuroprotection and antioxidant function following severe SCI. This combination synergistically enhanced motor function recovery, as quantitatively assessed by BBB scores and ladder-climbing tests. This functional improvement was accompanied by reduced accumulation of ROS and alleviation of neuroinflammation, as indicated by decreased microglial activation and glial scar formation, and increased distribution of 5-HT and NF-positive nerve fibers. The potent anti-inflammatory effects observed in our study warrant further discussion. We found that Lut-AST treatment significantly attenuated microglial activation, as shown by the reduced Iba1^+^ area and a distinct morphological shift from an activated, amoeboid state to a more ramified, surveillant phenotype. This finding is powerfully contextualized by the work of Lin *et al*., who demonstrated that AST monotherapy could suppress mTORC1 (Mechanistic Target of Rapamycin Complex 1) signaling to concomitantly regulate both microglia and neurons [43]. Our results suggest that the Lut-AST combination may potentiate this dual regulation. Furthermore, the significant downregulation of GFAP indicates an inhibition of reactive astrogliosis. This aligns with the recent findings of Rao *et al*., who reported that a drug combination containing AST could shift astrocyte polarization from a detrimental A1 state to a protective A2 state via the Sirt1–NF-κB pathway [44]. It is plausible that our combination therapy engages a similar mechanism to modulate the glial scar, thereby creating a more permissive environment for axonal growth. Concurrently, the antioxidant properties of the Lut-AST pair were a cornerstone of its efficacy. The significant reduction in ROS production, as visualized by DHE staining in the lesion epicenter, provides direct *in vivo* evidence of attenuated oxidative stress. This effect can be mechanistically linked to previous work, which specifically showed that Lut activates the Nrf2 antioxidant pathway in an SCI model [45]. It is highly likely that Lut’s potent Nrf2 activation within our combination works in concert with AST’s documented free-radical scavenging abilities, creating a synergistic antioxidant defense that mitigates secondary damage. Paterniti *et al*. investigated the neuroprotective effects of a co-ultramicronized composite of palmitoylethanolamide and Lut in a mouse model of SCI. Their study demonstrated that the combination of palmitoylethanolamide and Lut exerted potent anti-inflammatory and antioxidant effects, reducing cyclooxygenase-2 (COX-2) and inducible nitric oxide synthase (iNOS) expression and protecting against cell death [46]. Our results are consistent with these findings, as we also observed reduced expression of pro-inflammatory mediators and enhanced neuronal survival following Lut-AST treatment. However, our study extends these observations by demonstrating the synergistic effects of Lut and AST in promoting functional recovery and tissue repair, suggesting a broader therapeutic potential for this combination.

The co-administration of Lut and AST demonstrated superior outcomes compared to monotherapy in terms of neurological function recovery, antioxidant capacity, and inflammation suppression. Previous studies using Huangqi or Dangshen extracts in SCI models primarily employed oral or intraperitoneal administration, achieving systemic immunomodulation and mild neuroprotection, but failing to sustain therapeutic concentrations in spinal cord tissues [17]. In contrast, a single *in situ* injection of Lut-AST was selected to target the injured spinal cord in our study. Compared to systemic routes like oral gavage or intraperitoneal injection, a single local intralesional injection offers distinct advantages following SCI. Firstly, it ensures precise and immediate drug delivery to the lesion site, bypassing the blood–spinal cord barrier and achieving a high local concentration directly at the injury epicenter. This is crucial for effectively modulating the acute destructive cascade of secondary injury. Secondly, it minimizes systemic exposure and potential off-target side effects, thereby reducing the drug dose required and enhancing the therapeutic index. Systemic administration often results in significant first-pass metabolism (oral) or distribution to non-target organs, leading to lower bioavailability at the actual lesion site and a higher risk of systemic toxicity. In addition, compared to repeated injections, a single local injection minimizes repeated surgical trauma and stress to the animals, which themselves can exacerbate neuroinflammation and confound the interpretation of functional recovery data. We acknowledge that alternative delivery systems (e.g. sustained-release hydrogels) might offer distinct advantages for supporting longer-term neural remodeling and represent a highly valuable direction for our future work. However, given our study’s focus on investigating the initial regulatory effects of Lut-AST on early SCI pathology, the single *in situ* injection strategy was deemed the most appropriate to address our objective. We plan to explore multiple delivery methods in future work to further optimize Lut-AST’s therapeutic potential for SCI.

### Potential targets and synergistic mechanisms of AST-Lut treatment in SCI

Network pharmacology and molecular docking analyses identified five core targets through which Lut and AST exert synergistic effects on SCI: androgen receptor (AR), aurora kinase B (AURKB), checkpoint kinase 1 (CHEK1), caspase-3, and poly(ADP-ribose) polymerase 1 (PARP1). The AR is a critical transcription factor involved in the regulation of androgen-dependent genes, which play roles in pain and inflammation modulation [47, 48]. Our molecular docking studies revealed that Lut binds to AR at specific amino acid residues (Asn705, Gln711, Met745, and Phe764), suggesting the potential for Lut to activate AR and exert anti-inflammatory and wound repair-promoting effects. This aligns with prior studies showing that AR activation reduces glial scarring via inhibiting astrocyte overactivation [49], shifts microglia to the anti-inflammatory M2 phenotype [50], and upregulates neurotrophic factors (e.g. brain-derived neurotrophic factor (BDNF)) to improve motor function [51]. Consistently, our *in vivo* data demonstrated Lut-AST reduced Iba1 (microglia) and GFAP (astrocytes) expression, promoted NF expression, and elevated BBB scores, supporting AR-mediated neuroprotection.

AURKB is a serine/threonine protein kinase involved in mitotic regulation, and its overexpression has been implicated in various pathological conditions, including chronic pain and neuroinflammation. Our molecular docking studies suggest that Lut binds to AURKB’s kinase domain (residues His250, Arg248, Glu125) [52], inhibiting its catalytic activity. AURKB upregulation in SCI is linked to M1 microglial polarization, pro-inflammatory cytokine (tumor necrosis factor-alpha (TNF-α), interleukin-6 (IL-6)) release, and neuropathic pain [53]; it also phosphorylates nuclear factor of activated T-cells 5 (NFAT5) to enhance astrocyte swelling [54]. By inhibiting AURKB, Lut-AST treatment may attenuate microglial activation and reduce neuroinflammation, contributing to improved functional recovery. CHEK1 is a serine/threonine kinase involved in DNA damage response and cell cycle regulation. Our molecular docking results indicate that Lut targets CHEK1 (residues Glu55, Asn59, Lys38) [55], impairing its ATP anchoring and autophosphorylation. CHEK1 overexpression in SCI microglia drives ferroptosis (via inhibiting glutathione peroxidase 4 /xCT system (GPX4/x-CT)) and ROS accumulation [56, 57]. This suggests that Lut-AST treatment may reduce oxidative stress-induced neuronal damage by modulating the CHEK1 pathway, contributing to neuroprotection and functional recovery.

Caspase-3 is a key executor of apoptosis, and its activation is a hallmark of neuronal death in SCI. AST was shown to form hydrogen bonds with caspase-3’s Asn80 residue [58], stabilizing its structure and inhibiting self-activation. Caspase-3 upregulation in SCI exacerbates neuronal apoptosis [59]. AST has previously been shown to reduce caspase-3 via transcription factor EB (TFEB) activation [60]. Our data imply that Lut-AST inhibits caspase-3 through dual mechanisms: indirect suppression of inflammation/oxidative stress and direct structural stabilization by AST. Additionally, molecular docking results sugges that AST may bind to PARP1’s catalytic domain (residues Asn906, Tyr907) [61, 62], maintaining conformational stability. Excessive PARP1 activation in SCI triggers mitochondrial oxidative stress and apoptosis, while PARP1 inhibitors protect spinal cord tissue [63–65]. Our experimental results indicate that the Lut-Ast drug combination not only improves the pathological environment of the spinal cord in SCI rats and reduces the pathological activation and expression of PARP1 but may also directly bind to PARP1, maintaining its structural stability, thereby inhibiting cellular oxidative stress and apoptosis.

### Limitations and future directions

Despite the promising efficacy of Lut-AST combination reported in the present study, there still exist several limitations in our work. The *in vivo* functional assessment mainly focused on motor function, but lacked evaluations of sensory function and additional motor tests, which may have provided a more holistic functional recovery. We would include sensory function evaluations (e.g. von Frey test) and the inclined plane test in our future studies to address this limitation and provide a more comprehensive view of functional recovery. While sufficient to capture early-to-subacute-phase therapeutic effects of Lut-AST, the 5-week post-injury timeframe is shorter than the commonly recommended 8–12 weeks for assessing long-term tissue repair and functional sustainability. In future research, we will extend the observation period to 8–12 weeks to evaluate the long-term functional and histological outcomes of Lut-AST therapy. The scope of our neural regeneration analysis was limited to NF and 5-HT, while it lacked several key markers such as neuron-specific class III beta-tubulin (Tuj-1), nestin, and neuronal nuclear antigen (NeuN), which are valuable for comprehensively elucidating neural regeneration. In the subsequent study, we would incorporate these markers to precisely delineate the effects of Lut-AST on different stages of neuronal differentiation and maturation. We demonstrated Lut-AST’s anti-inflammatory effects via Iba1 staining, which demonstrated significant reduction in Iba1^+^ area and morphological transformation of microglia from an amoeboid state to a more ramified state. Nevertheless, CD68 and mRNA quantification of pro-inflammatory factors (e.g. IL-6, TNF-α) would further support neuroinflammation modulation of Lut-AST and offer additional molecular detail. Validating these specific molecular targets will be a primary focus of our immediate follow-up study. Additionally, our *in vitro* experiments were conducted in the PC12 cell line rather than in primary neuronal cultures that better mimic *in vivo* neuronal microenvironments. Future work would confirm these findings in primary neuronal cultures to confirm the neuroprotective and antioxidant effects of Lut-AST. Finally, our network pharmacological analysis identified potential therapeutic targets and key signaling pathways of Lut-AST in SCI. This analysis provides a theoretical framework and preliminary guidance for our subsequent *in vivo* and *in vitro* experiments. However, current work lacked direct molecular validation of the predicted pathways and core targets such as CASP3, PARP1, and AURKB. To address this limitation, we would validate the involvement of the top-predicted signaling pathways and targets via western blot, ChIP-qPCR, and specific pharmacological inhibition/activation studies in our future research plan. The validation of these predicted targets/pathways would further strengthen the causal link between Lut-AST’s mechanism of action and the network pharmacology findings.

Future research should further explore synergistic regulatory mechanisms of Lut-AST by spatial transcriptomics and phosphoproteomics [66], which could delineate the interplay between Lut-AST and key pathways such as Nrf2/GPX4 (ferroptosis), TFEB/mTORC1 (autophagy-lysosomal regulation), and NF-κB/NLRP3 (neuroinflammation). Additionally, temporal control of drug release is critical and advanced delivery systems (such as ROS-responsive nanoparticles or injectable hydrogels) could optimize pharmacokinetic profiles by enabling sequential drug release: initial Lut burst for acute oxidative stress mitigation, followed by sustained AST delivery to support chronic neural remodeling [67, 68]. In addition, technologies such as artificial intelligence will also significantly enhance the efficiency of the screening process [69, 70]. Such engineered formulations may amplify therapeutic efficacy while minimizing off-target effects, ultimately bridging the gap between TCM-inspired combinatorial therapy and precision medicine for SCI rehabilitation [71–73].

In conclusion, our study provides compelling evidence for the multi-target therapeutic effects of the Lut-AST combination in promoting neural repair and functional recovery after SCI. The involvement of AR, AURKB, CHEK1, caspase-3, and PARP1 pathways highlights the complex molecular mechanisms underlying the neuroprotective and regenerative effects of this combination. These findings, together with insights from relevant studies, support the potential of Lut-AST as a novel and effective strategy for SCI management. The Lut-AST combination represents a paradigm shift in SCI therapy, merging TCM's multi-target philosophy with modern precision medicine. By optimizing spatiotemporal drug delivery and elucidating synergistic mechanisms, this strategy holds transformative potential for neural repair.

## Supplementary Material

pbaf037_Supplemental_File
