# Supplementary material for: Drug pair-derived synergistic therapy of flavonoids luteolin and astragaloside IV promotes neural repair following spinal cord injury via antioxidant and neuroprotective effects
Source: Precis Clin Med. 2025 Dec 18;9(1):pbaf037. doi: 10.1093/pcmedi/pbaf037 (PMC12856357; doi:10.1093/pcmedi/pbaf037)
Supplement: pbaf037_Supplemental_File [file pbaf037_supplemental_file.docx]

**Supplementary materials**

**Images related to the SCI protocol application, tissue sampling, and BBB evaluation procedures are shown as follows:**

**1. SCI surgery**


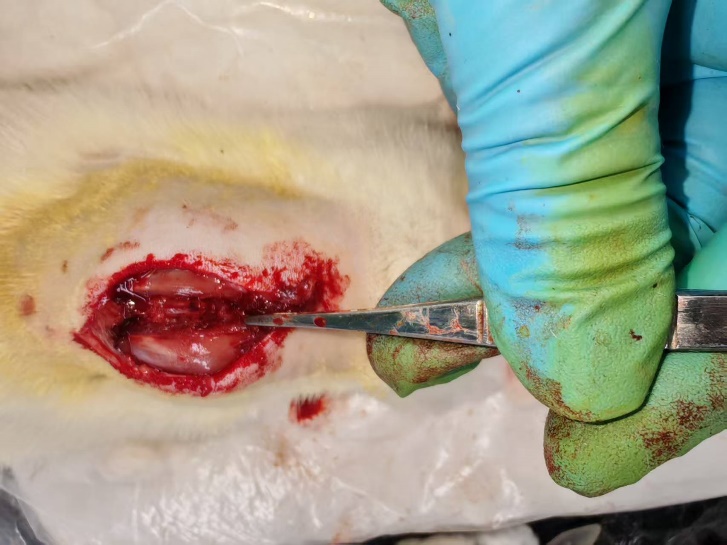


**2. Tissue harvesting 3. BBB scoring**


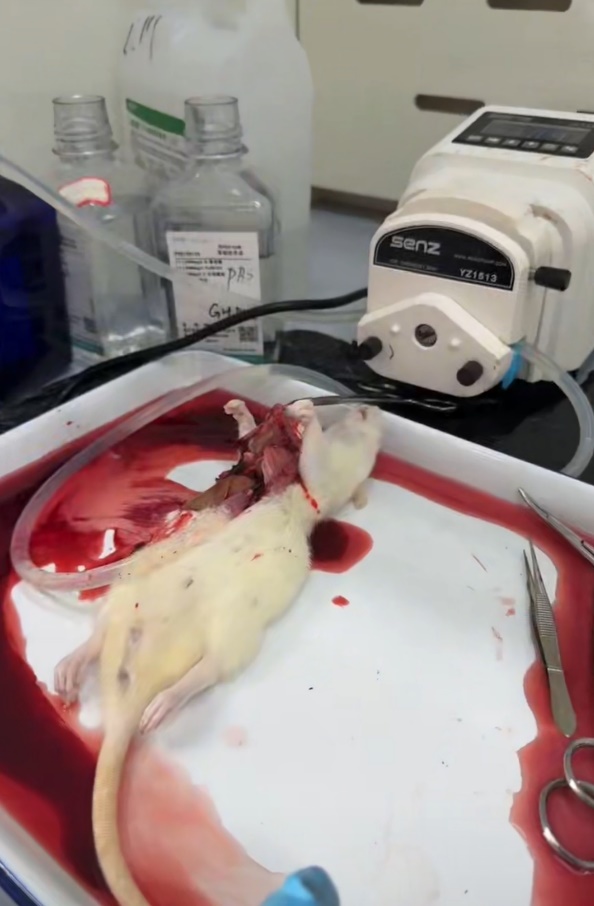

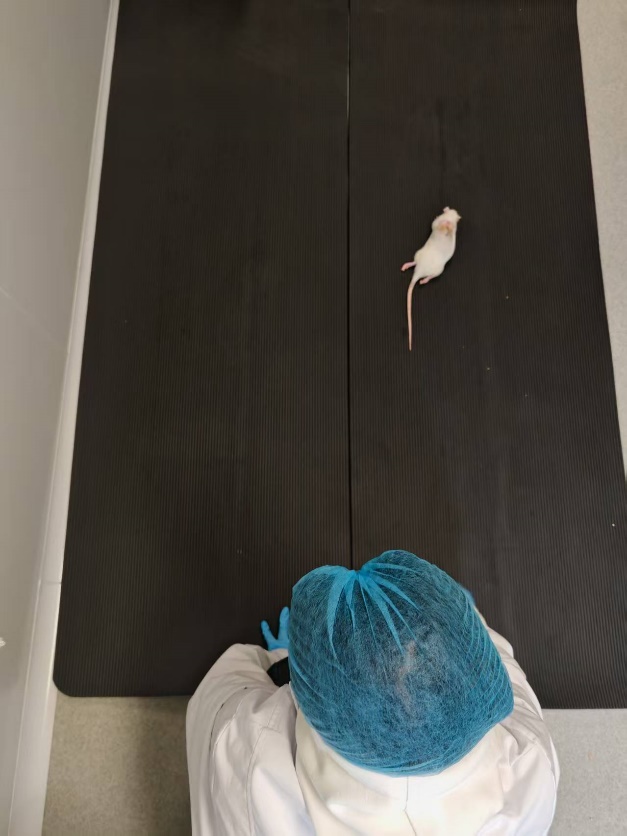


**Supplementary Table 1. BBB Rating Scale**

| Scores 0-7: Primarily evaluate isolated joint movements of the hindlimbs following spinal cord injury. | |
| --- | --- |
| 0 | No observable hindlimb movement. |
| 1 | Slight movement(s) of one or two joints, typically the hip and/or knee. |
| 2 | Extensive movement of one joint OR extensive movement of one joint and slight movement of another joint. |
| 3 | Extensive movement of two joints. |
| 4 | Slight movement of all three hindlimb joints (hip, knee, ankle). |
| 5 | Slight movement of two joints and extensive movement of the third joint. |
| 6 | Extensive movement of two joints and slight movement of the third joint. |
| 7 | Extensive movement of all three hindlimb joints. |
| Scores 8-14: Focus on assessing gait parameters during the intermediate recovery phase, including weight-bearing and coordinated motion. | |
| 8 | Plantar placement of the paw with no weight support during stance. |
| 9 | Plantar placement with either occasional weight-supported stance OR dorsal stepping with no plantar stepping. |
| 10 | Occasional plantar stepping with no forelimb-hindlimb coordination. |
| 11 | Frequent to consistent plantar stepping, but no forelimb-hindlimb coordination. |
| 12 | Frequent to consistent plantar stepping with occasional forelimb-hindlimb coordination. |
| 13 | Frequent to consistent plantar stepping with frequent forelimb-hindlimb coordination. |
| 14 | Consistent plantar stepping, consistent forelimb-hindlimb coordination, OR consistent plantar stepping with consistent coordination and occasional dorsal stepping. |
| Scores 15-21: Focus on evaluating the recovery of fine motor control in the late phase after spinal cord injury. | |
| 15 | Consistent plantar stepping and consistent coordination. During forward limb advancement, the predominant paw position is parallel to the body at initial contact, with or without paw dragging. The tail is down or occasionally up. |
| 16 | Consistent plantar stepping and consistent coordination. Frequent paw dragging during forward advancement. Predominant paw position is parallel to the body at initial contact and rotates after weight support. The tail is occasionally up. |
| 17 | Consistent plantar stepping and consistent coordination. Frequent paw dragging during forward advancement. Predominant paw position is parallel to the body both at initial contact and during weight support. The tail is up most of the time. |
| 18 | Consistent plantar stepping and consistent coordination. Consistent paw dragging during forward advancement. Predominant paw position is parallel to the body at initial contact and rotates during weight support. The tail is up. |
| 19 | Consistent plantar stepping and consistent coordination. Consistent paw dragging during forward advancement. Predominant paw position is parallel to the body both at initial contact and during weight support. The tail is down or occasionally up. |
| 20 | Consistent plantar stepping, consistent coordination, and consistent toe clearance. Predominant paw position is parallel to the body throughout the step cycle, including initial contact and during weight support. The trunk is unstable. The tail is consistently up. |
| 21 | Consistent plantar stepping, consistent coordination, and consistent toe clearance. Predominant paw position is parallel to the body throughout the step cycle. The trunk is consistently stable. The tail is consistently up. |

**Supplementary Table 2. Key reagents**

| **Reagent** | **Product brand** | **Product code** |
| --- | --- | --- |
| Fetal Bovine Serum | PAN | ST30-3302 |
| Penicillin-Streptomycin Solution | Procell | PB180120 |
| RPMI-1640 Basal Medium | Procell | PM150110 |
| Hydrogen Peroxide Solution | AEX | HG612515 |
| Dimethyl Sulfoxide (DMSO) | Solarbio | D8371 |
| Sodium Pentobarbital | Merck | 4390-16-3 |
| Xylene | China National Medicines | 10023428 |
| Absolute Ethanol | Shanghai Laboratory | 10009265 |
| Hematoxylin and Eosin (H&E) Stain | Biosharp | BL735B |
| Neutral Balsam | Solarbio | G8590 |
| NF Antibody | Cell Signaling Technology | 2836S |
| GFAP Antibody | Proteintech | 16825-1-AP |
| Iba1 Antibody | Proteintech | CL594-10904 |
| 5-HT Antibody | Proteintech | 26438-1-AP |
| CoraLite488-conjugated Donkey Anti-Mouse IgG(H+L) | Proteintech | SA00013-5 |
| CoraLite594-conjugated Donkey Anti-Rabbit IgG(H+L) | Proteintech | SA00013-8 |
| CoraLite488-conjugated Donkey Anti-Rabbit IgG(H+L) | Proteintech | SA00013-6 |
| Antifade Mounting Medium | Solarbio | S2110 |
| Dihydroethidium (DHE) Working Solution | Biosharp | BL762A |
| Luteolin | Macklin | L812409-1g |
| Astraloside | Macklin | A928102-250mg |
| DCFH-DA | Solarbio | D6470-25 |
